# Unlocking Enol-Ugi-Derived Conformationally Restricted Peptidomimetic Motifs

**DOI:** 10.1021/acs.joc.5c00229

**Published:** 2025-03-13

**Authors:** José Luis Ramiro, Jesús Díaz, Ana G. Neo, Carlos F. Marcos

**Affiliations:** Laboratory of Bioorganic Chemistry & Membrane Biophysics (L.O.B.O.). Departamento de Química Orgánica e Inorgánica, Universidad de Extremadura, Cáceres 10003 Spain

## Abstract

The enol-Ugi condensation, a versatile multicomponent reaction, provides a rapid and efficient route to enamine peptidomimetics. In this study, we investigated the factors influencing the conformational behavior of three enol-Ugi adducts with distinct structural features. Through DFT calculations and NCI analysis, we identified that noncovalent interactions, including hydrogen bonds and π–π interactions, play a pivotal role in restricting conformational flexibility. While six-membered cyclic enamines **6** and **7** exhibited varying degrees of rotational freedom, the indanone-derived enamine **8** displayed a locked conformation resembling a retropeptidic turn. These findings highlight the potential of tailoring enol-Ugi adducts to mimic biologically relevant peptidic motifs, opening new avenues for drug discovery and design.

## Graphical abstract

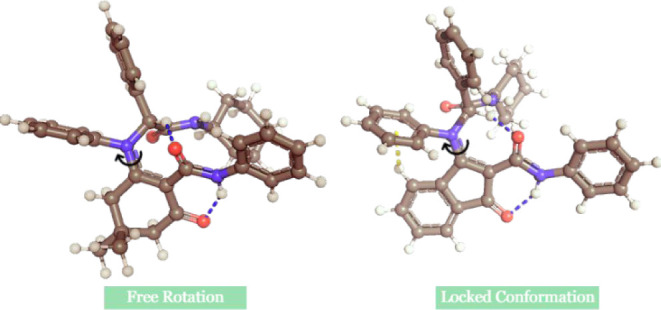

## Introduction

Peptidomimetics are synthetic molecules designed to emulate the three-dimensional structure and biological activity of natural peptides while overcoming their inherent limitations.^1^ These peptide analogues address the constraints of the parent compounds by exhibiting enhanced metabolic stability, favorable bioavailability, and improved receptor affinity and selectivity, often introducing conformationally restricted structural motifs. The restricted rotation around sigma bonds that results in the existence of rotational isomers or atropisomers is an advantageous characteristic for drug development, as it limits the number of conformations that can access pharmacological targets.^2^

Initially limited to biaryl compounds, atropisomerism has recently expanded to include C–N axially chiral molecules, such as diarylamines, which have become an important subject of research, notably for the development of enantiopure drugs, including conformationally restricted peptide mimics.^3^ However, the development of atropisomerically stable diarylamines is hindered by the possibility of a concerted rotation around two contiguous N–Ar bonds, which results in low energy barriers. To prevent this rotation and achieve stable conformations, bulky substituents, intramolecular hydrogen bonds or other constraints are often introduced.^4^ Notable examples of bioactive atropisomers include peptidomimetic γ-secretase inhibitor LY-411575 ^5^ (**1**) and Sotorasib (**2**),^6^ the first FDA-approved KRASG12C inhibitor, marketed as a single and stable C–N atropisomer. Furthermore, many diarylamines, such as the FDA-approved drugs Bosutinib (**3**), Mefenamic acid (**4**), and Imatinib (**5**) (Figure 1), are known to bind their respective targets in a single atropisomeric conformation.^3b^

**Figure 1:**
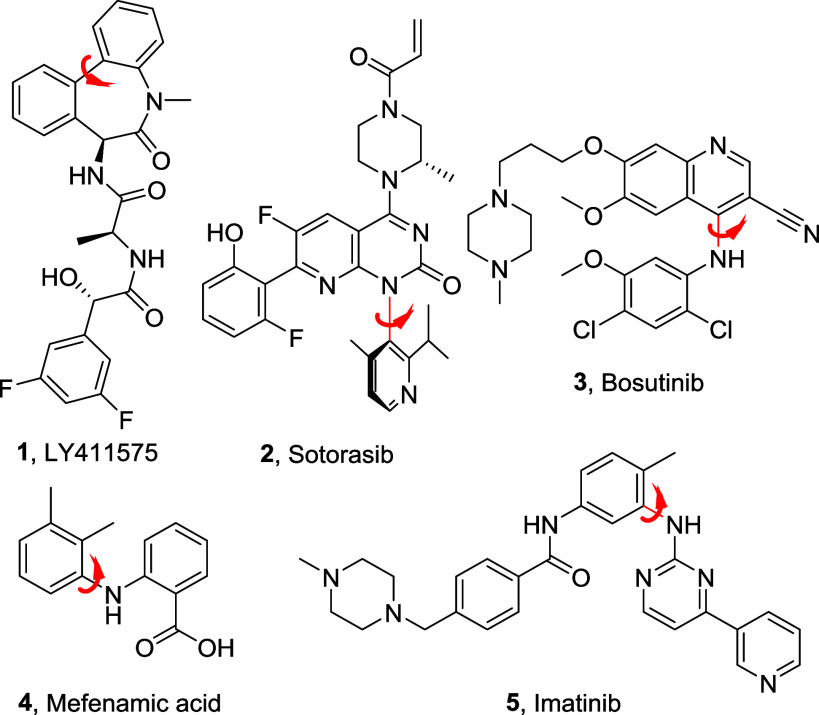
Drugs containing atropisomeric diarylamino motifs.

Different strategies have been devised to create conformationally restricted molecular scaffolds that mimic peptide secondary structures. Among these approaches, isocyanide-based multicomponent reactions, such the Passerini^7^ and the Ugi^8^ condensations and their variants, stand out as particularly advantageous. These reactions can generate complex peptidic structures in one-pot with a high efficiency and atom economy.^9^ Furthermore, they have been extensively used for the synthesis of peptidomimetics, usually in combination with postcondensation transformations that result in small ring constraints or macrocyclic structures.^10^

The enol-Ugi reaction, a variant of the classical Ugi four-component condensation developed in our laboratory, offers a direct route to synthesizing β-enamine peptidomimetics.^11^ By judiciously selecting starting materials or employing postcondensation transformations, the enol-Ugi reaction can be tailored to incorporate turn-inducing motifs with constrained conformations. These motifs are essential structural elements for designing peptidomimetics that feature antiparallel^12^ or parallel^13^ β-strands.

Despite recent investigations into the rotational dynamics of axially chiral enamides,^14^ no reports of atropisomeric tertiary aryl enamines have been published to our knowledge. Understanding how restricted rotation around *N*-aryl and *N*-enamine bonds can result in the formation of stable conformations could provide valuable insights into the design and development of functional molecules based on the axially chiral enamine scaffold.

This study utilizes computational Density Functional Theory (DFT) methods to elucidate the conformational landscape of three dipeptide mimics (**6**–**8**; Figure 2) with varying degrees of conformational flexibility, synthesized via enol-Ugi reactions. Our goal is to identify structural motifs that govern the geometrical restrictions around specific bonds in these molecules and correlate them with their NMR spectroscopic properties, using both experimental and computational approaches.

**Figure 2:**
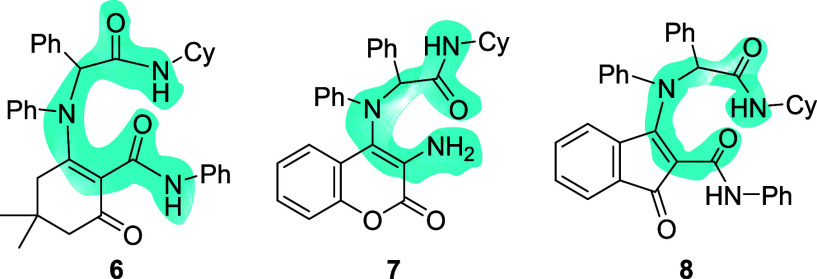
Peptidomimetic enamines obtained by enol-Ugi condensations.

The results of this work will provide valuable information for the design of peptidomimetics containing conformationally stable turn motifs. The modular nature of the enol-Ugi reaction offers opportunities to extend both ends of the enol-Ugi adducts, enabling the construction of larger peptidomimetic sequences. Modification of the isocyanide end, a well-established strategy in multicomponent reaction chemistry, allows for the introduction of various functional groups and peptide chains.^15^ For instance, Rivera and Wessjohann employed convertible isocyanides in the ligation of multicomponent reaction-derived peptides, yielding lineal and cyclic peptides.^15c^ Peptide-derived isocyanides have also been used to introduce peptidic chains into enol-Ugi adducts.^16^ The enol end can also be modified by employing enol-amides with functionalizable or cleavable groups in the enol-Ugi condensation. These enol-amides are readily accessible through reported procedures.^17^

## Results and Discussion

The synthesis of nonpeptide peptidomimetic enamines **6**–**8** (Figure 2) was recently reported.^18^ Enamines **6** and **8** were obtained through enol-Ugi reactions of cyclohexyl isocyanide and *N*,1-diphenylmethanimine with, respectively, 2-hydroxy-4,4-dimethyl-6-oxo-*N*-phenylcyclohex-1-ene-1-carboxamide and 3-hydroxy-1-oxo-*N*-phenyl-1*H*-indene-2-carboxamide. Enamine **7** was similarly synthesized by the enol-Ugi reaction of 4-hydroxy-3-nitrocoumarin followed by reduction of the nitro group.

Adduct **6** ^18a^ is expected to exhibit some conformational rigidity due to the presence of a six-membered ring and an intramolecular hydrogen bond between the phenylamide NH and the ketone carbonyl within the ring. A conformational search using density functional theory (DFT) calculations generated 4375 conformers. Setting a cutoff value of 10 kcal/mol identified the two most stable conformations for adduct **6** (**6A** and **6B** in Figure S9), which feature a retropeptidic turning motive stabilized by similar noncovalent interactions. However, the presence of a single set of signals in the NMR spectrum suggests rapid interconversion between these conformers in the NMR time scale.

The most stable conformation exhibits an uncommon hydrogen bond between the C=O group of the amide on the 6-membered ring and the proton on the stereogenic carbon, as revealed by noncovalent interaction (NCI) calculations (Figure 3).^19^ This hydrogen bond, likely resulting from the relative acidity of this proton,^20^ explains the anomalous chemical shift of 6.29 ppm in the ^1^H NMR spectrum.^21^ The stereogenic proton signal is substantially downfield compared to analogous protons in other enol-Ugi adducts (Figure 4).^11,22^ DFT calculations, weighed by the contributions of the most stable conformations and corrected applying Tantillo’s scaling factors based in experimental and theoretical data,^23^ yielded a chemical shift of 6.29 ppm, in excellent agreement with the experimental value. Interestingly, similar C­(sp^3^)­H···O hydrogen bonds have been reported to play a role in the stabilization of β-sheets in polypeptides.^24^ This suggests a potential link between the unusual conformation observed in adduct **6** and structural features commonly found in proteins. It is noteworthy that large downfield shifts of C-α hydrogen NMR signals, similar to those observed for enamine **6**, have been correlated with proximity to amide oxygen in protein antiparallel β-sheets^25^ and β-turn mimetics.^26^

**Figure 3:**
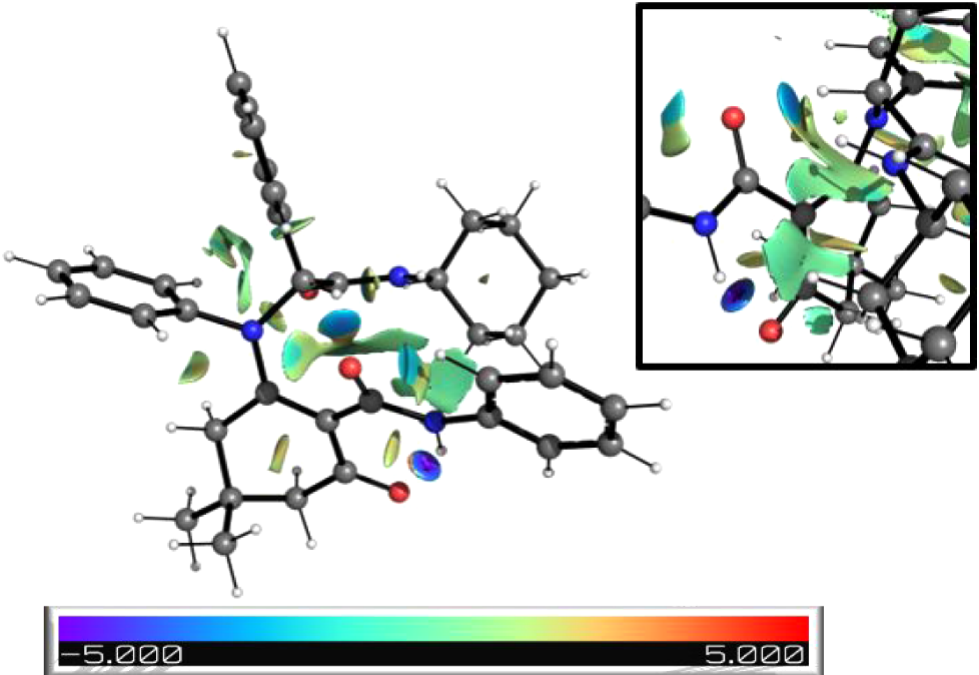
Most stable conformation of adduct **6** showing NCI calculations. Blue and red regions indicate attractive and repulsive interactions, respectively.

**Figure 4:**
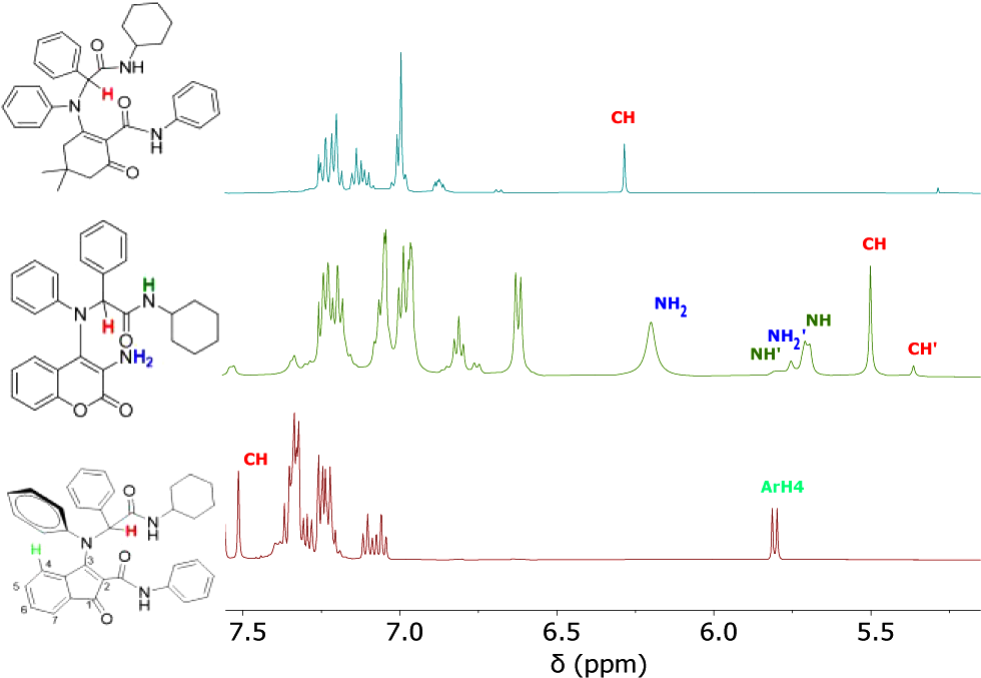
NMR spectra of adducts **6**–**8** showing the signals corresponding to the proton on the stereogenic carbon.

In pursuit of a more conformationally restricted analogue, benzopyran **7**, a conformationally hindered enamine, was synthesized as previously described by an iron reduction of the corresponding enol-Ugi adduct derived from 3-nitro-4-hydroxycoumarin, benzaldehyde, aniline and cyclohexyl isocyanide.^18b,18c^ The combination of the bulkier coumarin system and the hydrogen bond donor NH_2_ group introduces additional geometric constraints within the molecule.

Not surprisingly, the ^1^H NMR spectrum of compound **7** reveals the presence of two distinct rotational isomers at room temperature. Although these rotamers cannot be isolated individually, the rotational barrier impedes their free interconversion at room temperature, leading to the appearance of two distinct sets of signals in the ^1^H NMR spectrum in CDCl_3_ (Figure 4). These signals are evident for the protons on the amide NH (5.70 and 5.75 ppm) and on the stereogenic carbon (5.50 and 5.36 ppm). Integration of these peaks allows determination of the ratio of the two species as 90:10.

Variable temperature NMR experiments in Cl_4_C_2_D_2_ (Figures 5 and S1) revealed a coalescence temperature (*T*_c_) of 373.15 K, indicating the energy barrier for interconversion between the diastereomeric rotamers. Using the Eyring equation modified for unequal populations,^27^ the rotational barrier was calculated to be 18.33 kcal/mol for the conversion from the most stable to the least stable rotamer, and 17.04 kcal/mol in the opposite direction (see Supporting Information).

**Figure 5:**
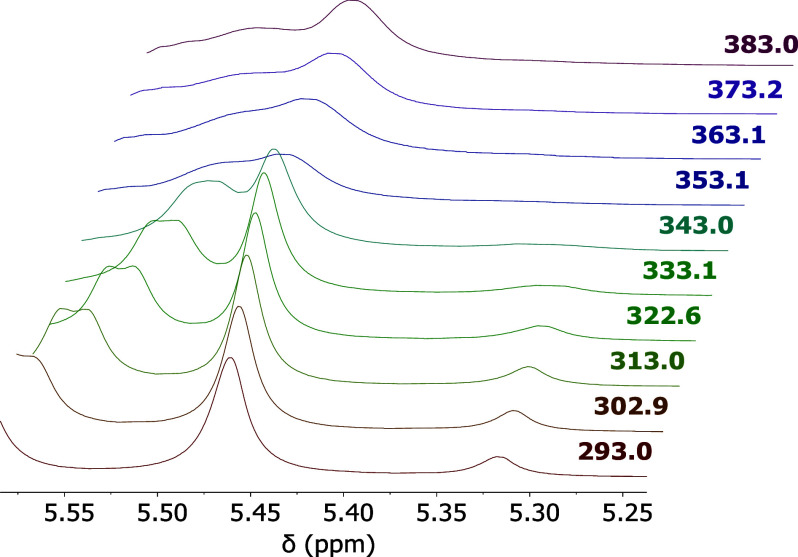
NMR spectra of **7** in Cl_4_C_2_D_2_ acquired at different temperatures, zoomed to show the signal corresponding to the proton on the stereogenic carbon.

Significantly, ^1^H NMR spectra of **7** recorded in polar solvents like DMSO, methanol or acetone do not exhibit evidence of diastereomeric rotamers, suggesting that interaction with these solvents disrupts the intramolecular hydrogen bonds crucial for stabilizing the restricted conformations in **7** (Figure S3). This is further supported by the single set of NMR signals observed for the adduct with a nitro group replacing the amine group on the coumarin position 3, where a hydrogen bond is not possible (**9**; Figures 6 and S6).^22b^

**Figure 6:**
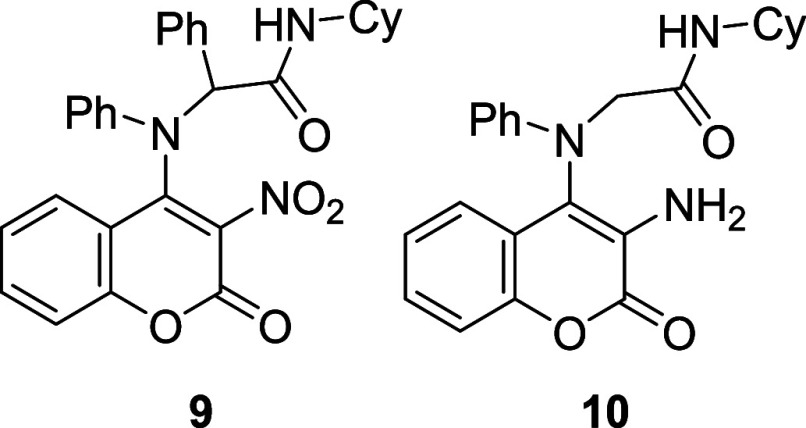
Coumarin enol-Ugi adducts **9** and **10**.

Interestingly, the ^1^H NMR spectrum of the formaldehyde adduct (**10**; Figure 6),^18b^ a less sterically hindered compound, showed two doublets corresponding to the diastereomeric protons of the CH_2_ next to the amide group, indicating the presence of rotamers. Variable temperature NMR experiments for this adduct yielded a Tc of 343 K, corresponding to a rotational barrier of 16,29 kcal/mol (Figure S2). Surprisingly, even in polar solvents like DMSO, acetone and methanol, the ^1^H NMR spectra of this adduct still exhibited distinct signals for the diastereomeric protons, with coalescence only observed upon the addition of formic acid to the CDCl_3_ solution (Figure S4). These findings suggest that the formaldehyde adduct (**10**) forms a stronger hydrogen bond than compound **7**, despite exhibiting a lower rotational barrier.

To achieve a more thorough understanding of the origin of the observed atropisomerism, we performed an extensive conformational search on both compounds **7** and **10** using DFT calculations. The dihedral angles primarily investigated for compound **7** were those responsible for breaking or maintaining the potential hydrogen bond between the amine NH and the carbonyl oxygen, i.e., the rotation around enamine-N (ϕ_1_), N–CHPh (ϕ_2_), and C–C­(=O)­NH (ϕ_3_) bonds represented with curved colored arrows in Figure 7.

**Figure 7:**
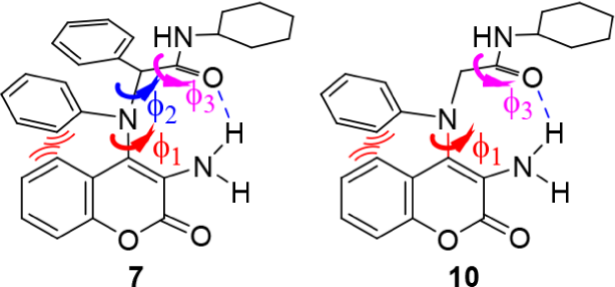
Structures of benzopyran adducts **7** and **10**, illustrating the bonds subjected to rotation in the conformational search.

Derived from this conformational search, we were able to identify an absolute minimum, and a second local minimum between more than one hundred conformations, both of them displayed in Figures 8 and S10. As predicted, the occurrence of two diastereomeric rotamers in **7** arises from a strong hydrogen bond between the NH_2_ group and the amide carbonyl oxygen, which enforces a pseudo-eight-membered ring system (Figure 7). This, along with the steric hindrance caused by the coumarin and the bulky substituents on the tertiary amine nitrogen, restricts the rotation around the bond between the nitrogen and the carbon 4 of the coumarin. Consequently, the amide group occupies opposite sides of the coumarin plane: on the left in the most stable conformation and on the right in the alternative conformer (Figure 8, zoomed regions). Figure 8 also shows the most significant noncovalent interactions identified through DFT calculations for both conformers. Predicted rotational barriers for compound **7** were 18.91 and 17.63 kcal/mol, respectively, which are in good agreement with the experimental values obtained from the variable temperature NMR experiments (18.33 and 17.04 kcal/mol, respectively). Notably, the conformations corresponding to the maxima of both rotational barriers do not allow the formation of an intramolecular hydrogen bond, emphasizing its crucial role in stabilizing the atropisomeric conformations (see Figure S11).

**Figure 8:**
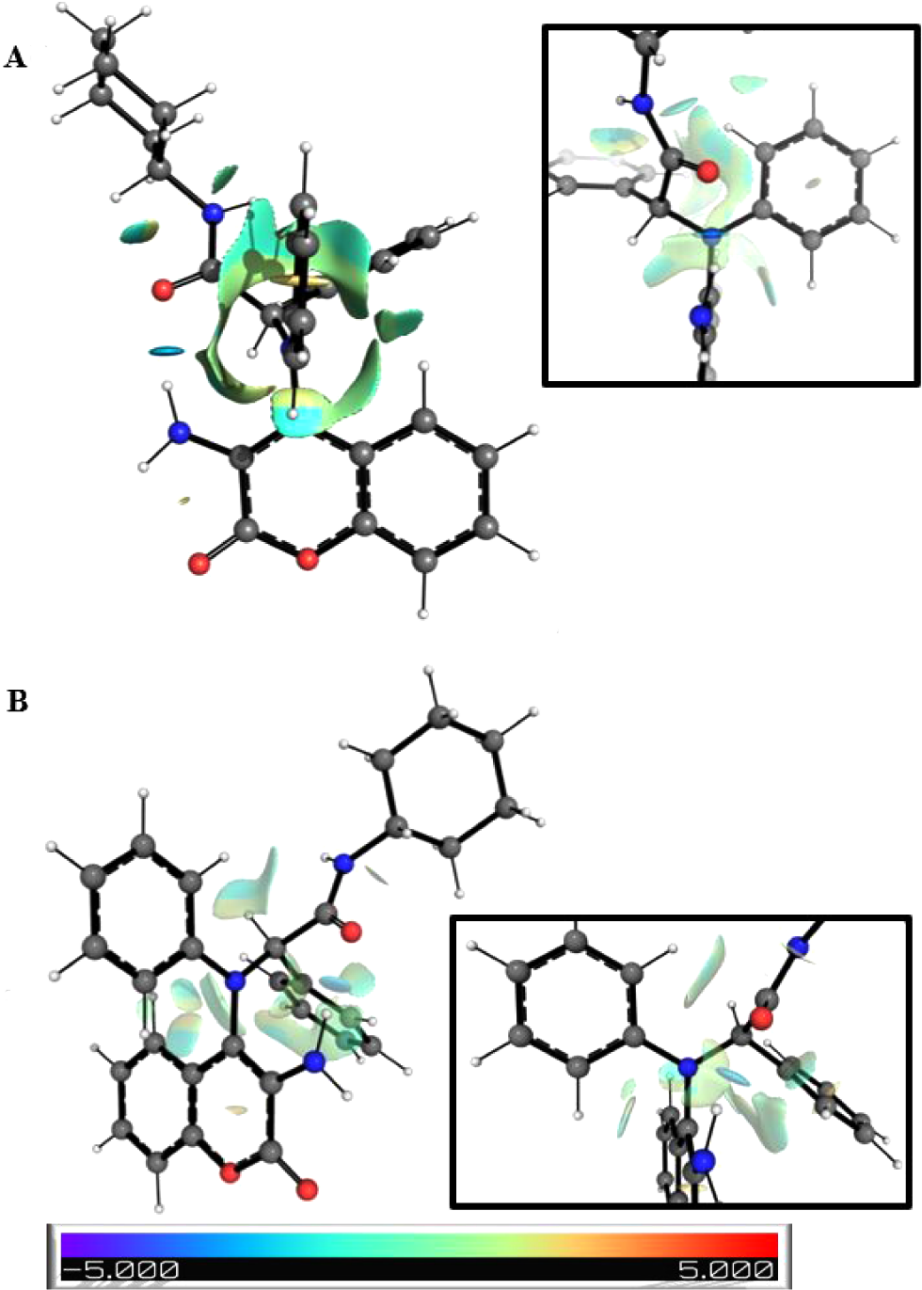
NCI plot for the two more stable computed conformers of **7** (**A**: most stable conformer, **B**: alternative conformer). Blue and red regions indicate attractive and repulsive interactions, respectively.

Computational calculations on the formaldehyde adduct (**10**) identified two enantiomeric low energy conformations separated by a theoretical rotational barrier of 16.34 kcal/mol (Figure S12). This value closely matches the barrier of 16.29 kcal/mol obtained from the VT NMR experiments. Due to reduced steric hindrance in compound **10** compared to **7**, the rotational scan focused on only two dihedral angles, ϕ_1_ and ϕ_3_ (Figure 7). A key difference between compounds **7** and **10** is that, while weaker than in the energy minima, some degree of hydrogen bonding between the NH_2_ and amide carbonyl oxygen is maintained at the rotational barrier maximum in compound **10** (Figure S12). This hydrogen bond, along with **10’**s less bulky structure, likely contributes to its lower rotational barrier. However, the hydrogen bond is demonstrably weaker at the rotational barrier maximum, as evidenced by a longer H···O distance (1.924 Å compared to 1.872 and 1.901 Å in the two most stable rotamers, respectively) and a lower NBO-calculated stabilization energy (2.95 kcal/mol compared to 9.30 kcal/mol in the most stable conformer; see Table 1). Therefore, polar solvents, which could preferentially disrupt this weakened hydrogen bond at the barrier maximum, may exert only a minimal influence on the rotational barrier. In contrast, formic acid, capable of disrupting hydrogen bonds in both the ground state and at the barrier maximum, effectively lowers the rotational barrier and precludes the observation of rotamers.

**Table 1:** NBO-Calculated Energy Stabilization for Hydrogen Bond in Compounds **7** and **10** ^a^

Compound	NBO donor	NBO acceptor	*E* (kcal/mol)
**7**	LP (O)	BD* N–H	4.48
**7**	LP (O)	BD* N–H	8.70
**10**	LP (O)	BD* N–H	5.94
**10**	LP (O)	BD* N–H	9.30

a LP: lone pair of oxygen; BD*: N–H antibonding orbital. The energy values represent the interaction between the N–H antibonding orbital and the two lone electron pairs of the carbonyl oxygen. The higher energy (most stabilizing) values correspond to the interaction with the lone pair that is optimally oriented for hydrogen bond formation.

Natural Bond Orbital (NBO) analysis^28^ was performed on both compounds **7** and **10** to gain deeper insights into the differences observed in their geometries and the hydrogen bond interactions between the amide group and the amino group. Table 1 presents the energy stabilization resulting from the transfer of electronic density from the hydrogen bond donor orbital (oxygen lone pairs, LP) to the acceptor orbital (N–H antibonding orbital, BD*). These energies provide an indirect measure of the hydrogen bond strength. The calculated difference in stabilization energies indicates a stronger hydrogen bond interaction in compound **10** than in compound **7**, which is consistent with the experimental observation that a more polar solvent, such as formic acid, is needed to disrupt the hydrogen bond in compound **10**. This can be attributed to several factors including steric hindrance in compound **7**, which prevents an optimal geometry, distorting the pseudo-sp^2^ nitrogen angle from the ideal 120° to 115°. In contrast, the corresponding nitrogen in compound **10** maintains a near-ideal 119° angle, allowing an optimal overlap between the donor lone pair (LP) and the acceptor antibonding orbital (BD*) and thus maximizing the stabilization energy (see Figure S13). This optimal geometry in compound **10** also results in a shorter donor–acceptor distance, which typically correlates with higher stabilization energies. While both compounds form a pseudo-eight membered ring geometry due to hydrogen bonding, compound **10** is characterized by reduced steric and angle strain compared to that of compound **7**. On the other hand, the lower steric bulk in compound **10** likely contributes to its lower rotational barrier. Therefore, both hydrogen bonding and steric hindrance, working in concert, appear to be responsible for the observed atropisomerism in these compounds.

With the aim of obtaining an axially chiral enamine with a locked turn conformation, indanone **8**, incorporating features from both adducts **6** and **7**, was synthesized via an enol-Ugi condensation of 2-amidoindandione.^18d^ This adduct exhibits a single set of signals in the ^1^H NMR spectra, indicating no atropoisomeric behavior. Furthermore, intriguing spectroscopic features are evidenced. The H4 aromatic proton of the indanone ring displays an unusual upfield shift of about 2 ppm compared to similar adducts with aliphatic substituents on the nitrogen, appearing as a doublet at 5.81 ppm.^18d^ Conversely, the signal of the proton on the asymmetric carbon is downfield-shifted by approximately 2 ppm compared to analogous protons in other enol-Ugi adducts,^11,22^ residing within the aromatic proton region at 7.51 ppm (Figure 4). This downfield shift was already observed for enamine **6** but is much more pronounced in this case, suggesting a stronger C­(sp^3^)­H···O hydrogen bond that locks the conformation in solution.^21,26^ Bidimensional COSY and HSQC NMR experiments of compound **8** unequivocally confirmed the assignment of these NMR signals. In the COSY spectra, it can be observed that the doublet at 5.81 ppm is coupled with an aromatic triplet, suggesting its aromatic character, while the signal at 7.48 ppm is not coupled with any other proton. This is further supported by the HSQC spectra, where a CH signal is correlated with the singlet at 7.48 ppm and the upfield doublet is directly bonded to a primary aromatic carbon (Figures S7 and S8).

A conformational search allowed us to obtain the DFT-calculated chemical shifts for H4 and the proton on the stereogenic carbon, weighed by the contributions of the most stable conformations. This procedure generated 5000 conformers, of which only those within 10 kcal/mol of the global minimum were analyzed. NMR chemical shifts were calculated using the scaling factors determined by Tantillo and coworkers,^23^ following a similar approach as for compound **6**. The calculated values were 5.67 and 7.83 ppm, respectively, in close agreement with the experimental values (5.81 and 7.48 ppm).

DFT calculations identified the most stable conformation of compound **8**, while NCI analysis revealed robust intramolecular noncovalent interactions stabilizing this conformation (Figure 9). The orientation of the amide group on the 5-membered ring is fixed by a hydrogen bond between the amide NH and the indanone carbonyl group. A second hydrogen bond between the C=O of this amide group and the proton on the stereogenic carbon forces the molecule to adopt a retropeptidic bent conformation similar to the most stable conformation of adduct **6**.^19b,21^

**Figure 9:**
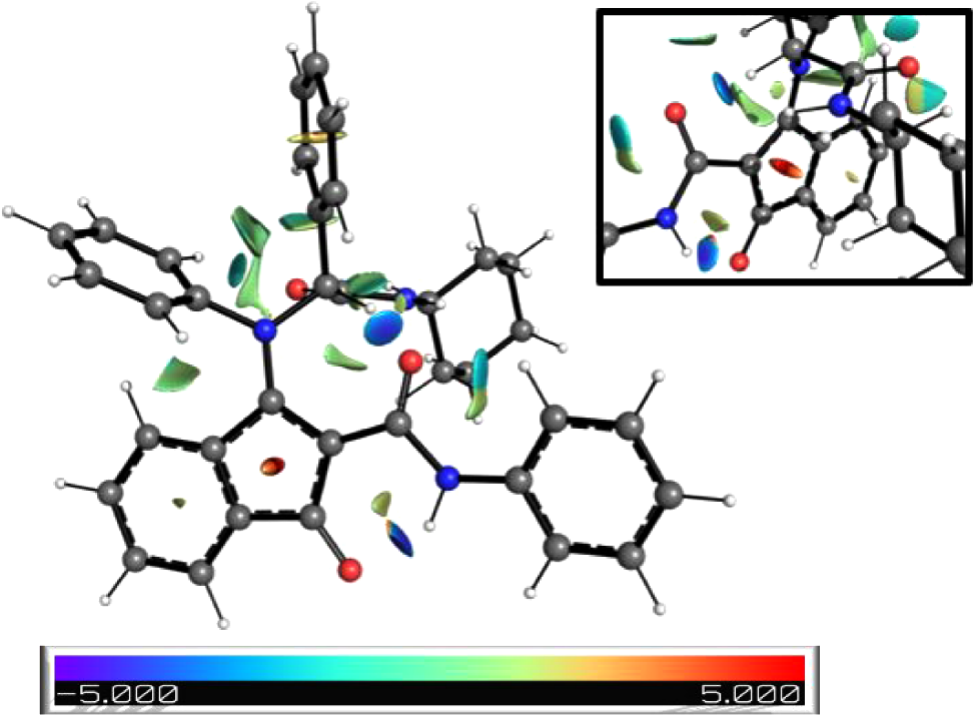
Gaussian minimized structure of adduct **8** and NCI calculations.

The deshielding effect of this H-bond on the NMR signal corresponding to the proton on the stereogenic carbon is significantly larger than in adduct **6**, suggesting a shorter distance and a stronger interaction as it can be observed in Figure 9 (see also Figure S9). This strong interaction persists even in polar solvents like acetone, methanol and chloroform with formic acid, as evidenced by the lack of significant changes in the NMR spectra (Figure S5). Maintaining a stable conformation in polar solvents is an important characteristic of pharmaceuticals, as it ensures the active conformation is present in the aqueous physiological media. NCI analysis further identified an edge-to-face π-π interaction between the indanone aromatic ring and the phenyl substituent on the amine group, which contributes to the shielding effect observed on the indanone H4 proton.

The synergistic influence of these two hydrogen bonds and the π-π interaction effectively locks the molecule in the conformation observed in ^1^H NMR spectra, corroborating the experimental findings. This spatial arrangement is facilitated by the relative positioning of the tertiary amine phenyl substituent and the indanone aromatic ring, allowing for the key interaction between the amine phenyl substituent and indanone H4. Similar edge-to-face π-π interactions have been observed in other enamino indanones.^29^ In contrast, coumarin adduct **7** lacks the necessary space for such an interaction due to its enamine being located on a six-membered ring instead of a five-membered ring. This larger ring structure prevents the amine phenyl substituent from getting close enough to the coumarin aromatic ring, leading to steric hindrance. This spatial incompatibility in compound **7** leads to restricted rotation around the *N*-enamine bond, resulting in two rotamers separated by a barrier and observed in NMR.

## Conclusions

In conclusion, this work provides valuable insights into the structural behavior and noncovalent interactions inherent to peptidomimetic enamines **6–8**, synthesized through enol-Ugi condensation. These features have been explored by computational DFT studies, which determined the most stable conformations, and NCI plots to identify the most relevant noncovalent interactions. Moreover, the computational estimation of anomalous chemical shifts for the protons involved in these interactions aligns with experimental NMR spectroscopy data.

Adduct **6**, exhibits a partially restricted rotation with its most stable conformation stabilized by a nonstandard C­(sp^3^)­H···O hydrogen bond, resembling those found in β-sheet protein structures. Despite this, rapid interconversion between conformers occurs in the NMR time scale, as evidenced by the NMR spectrum. Compound **7** demonstrated conformational restrictions, resulting in two distinct, nonisolable rotational isomers observable at room temperature in the ^1^H NMR spectrum. The presence of a strong intramolecular hydrogen bond is pivotal for the appearance of these conformers. Dynamic NMR studies and DFT calculations provide further understanding of the structure and rotational barriers between the conformers. Finally, the indanone derivative **8** presents a locked conformation stabilized by noncovalent interactions, including C­(sp^3^)­H···O and NH···O hydrogen bonds alongside a π-π interaction. These noncovalent forces produce a retropeptidic bent conformation similar to the most stable conformation of adduct **6**, which generates intriguing spectroscopic features, confirmed by DFT and experimental studies.

## Experimental Section

### General Experimental Protocols

^1^H, ^13^C NMR Temperature Variable ^1^H NMR experiment and bidimensional NMR spectra were acquired on a Bruker 500 MHz spectrometer. Chemical shifts (δ) are provided in parts per million (ppm). Synthesis procedures for compounds **6**, **7** and **8** were previously described.^18^

### Computational Methods

All calculations were performed with GAUSSIAN16.^30^ Geometries were optimized in the gas-phase using the B3LYP/6-31G­(d) level of theory.^31^ Frequency calculations (at 298.15 K) at the same level of theory were used to confirm the nature of all stationary points as minima and provided values for compute free energies. NMR single point calculations (GIAO)^32^ were performed on these geometries at the B3LYP+G­(d,p) level of theory with chloroform as solvent employing the Solvent Model Density (SMD) within the Self-Consistent Reaction Field method SCRF = (SMD, solvent = chloroform).^33^

## Supplementary Material



## Data Availability

The data underlying this study are available in the published article and its Supporting Information.

## Notes

^1^H NMR spectra of **7**, **8** and **10** at different temperatures, ^1^H NMR spectra of **7**, **8** and **10** in different solvents, COSY and HSQC spectra of **8**, calculation of rotational barriers from variable temperature NMR, computational calculations and XYZ matrices for computationally derived structures of compounds **6**, **7**, **8** and **10** (PDF)
