# Supplementary material for: Unlocking Enol-Ugi-Derived Conformationally Restricted Peptidomimetic Motifs
Source: J Org Chem. 2025 Mar 13;90(12):4382–8. doi: 10.1021/acs.joc.5c00229 (PMC12136727; doi:10.1021/acs.joc.5c00229)
Supplement: Supplementary file 1 [file jo5c00229_si_001.pdf]

Unlocking Enol-Ugi-Derived Conformationally Restricted Peptidomimetic motives

José Luis Ramiro, Jesús Díaz, Ana G. Neo and Carlos F. Marcos*

Laboratory of Bioorganic Chemistry & Membrane Biophysics (L.O.B.O.). Departamento de Química Orgánica e Inorgánica. Universidad de Extremadura. 10003 Cáceres, Spain.

Table of contents

General experimental protocols....................2

NMR spectra....................2

Calculation of the experimental rotational energy barriers....................10

Computational methods....................12

^{1}HNMR computed chemical shift....................12

Non-covalent Interactions (NCI) calculation....................13

Minima and maxima geometries for compounds **7** and **10**....................15

Matrices XYZ for computationally derived structures of compounds **6-10**....................17

References....................17

General experimental protocols

^{1}H NMR, ^{13}C NMR, Variable Temperature ^{1}HNMR experiments and bidimensional NMR spectra were acquired on a Bruker 500 MHz spectrometer. Chemical shifts (δ) are provided in parts per million (ppm). Synthesis procedures for compounds **6-10** were previously described.[1-3]

NMR spectra

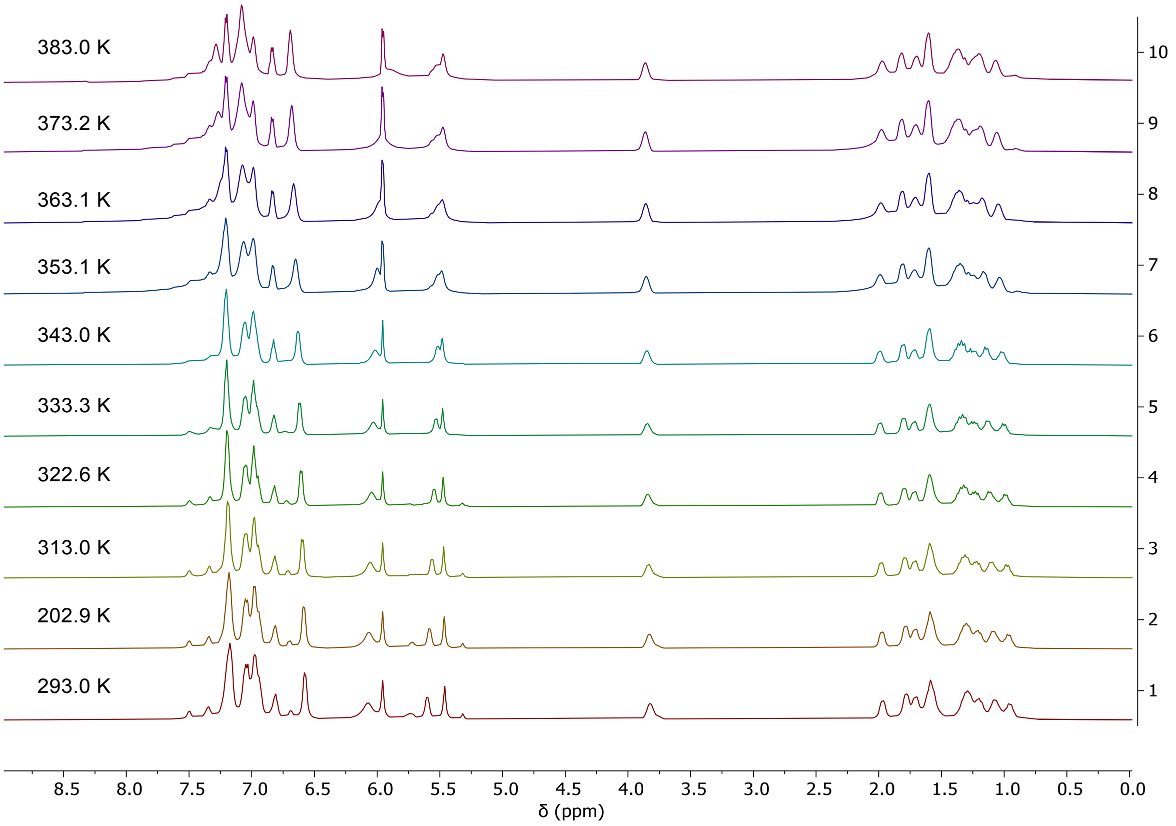

Figure S1. Stacked ^{1}HNMR spectra of compound **7** in 1,1,2,2-tetrachloroethane-*d2*, registered at increasing temperatures from 293 K to 383 K.

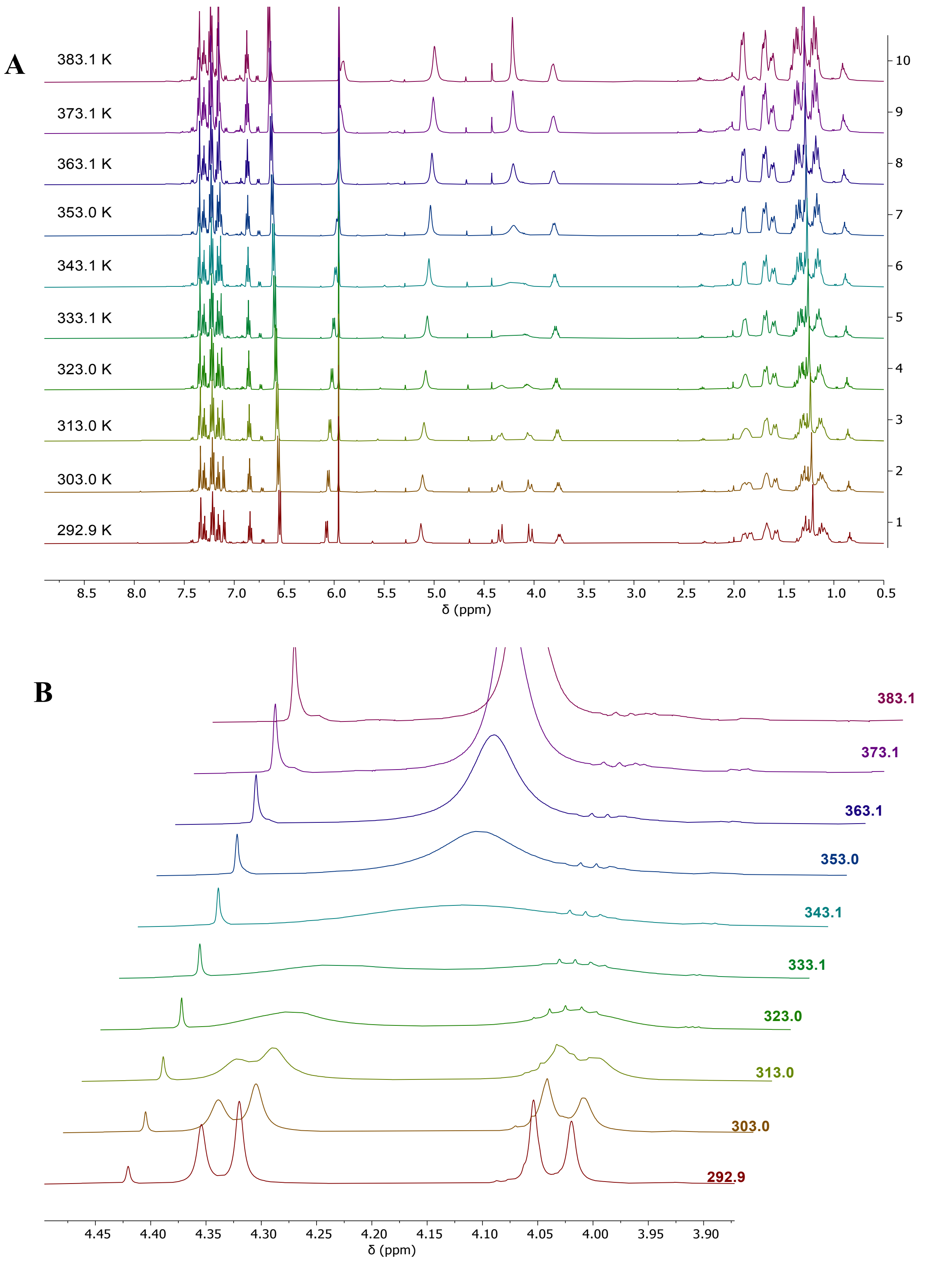

Figure S2. **A**. Stacked ^{1}HNMR spectra of compound **10** in 1,1,2,2-tetrachloroethane-*d2*, registered at increasing temperatures from 293 K to 383 K. **B**. Expansion of the area showing the signals corresponding to the diastereotopic protons.

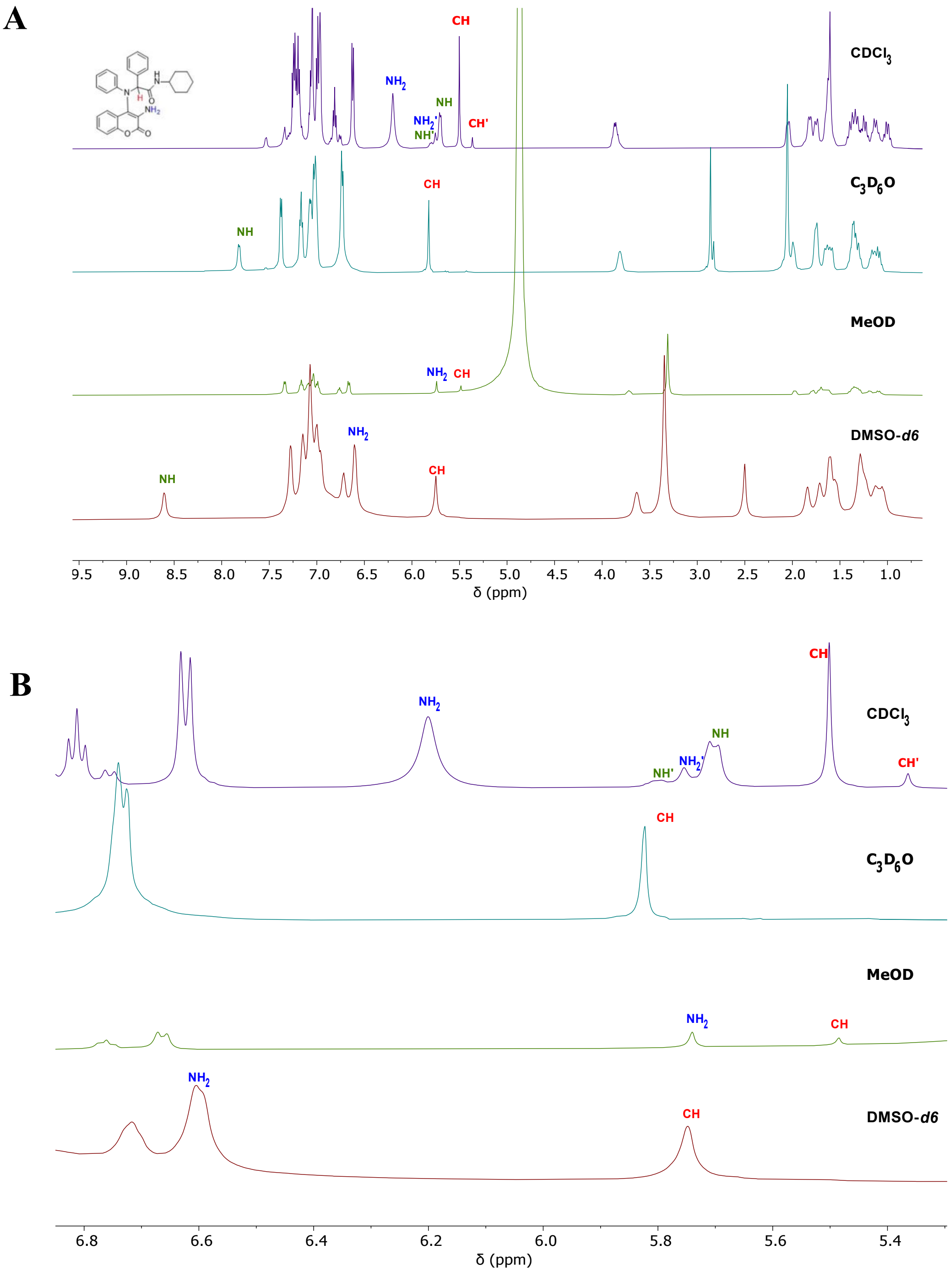

Figure S3. **A**. Stacked ^{1}HNMR spectra of compound **7** in different solvents. **B**. Expansion of the area showing the signals corresponding to the proton on the stereogenic carbon.

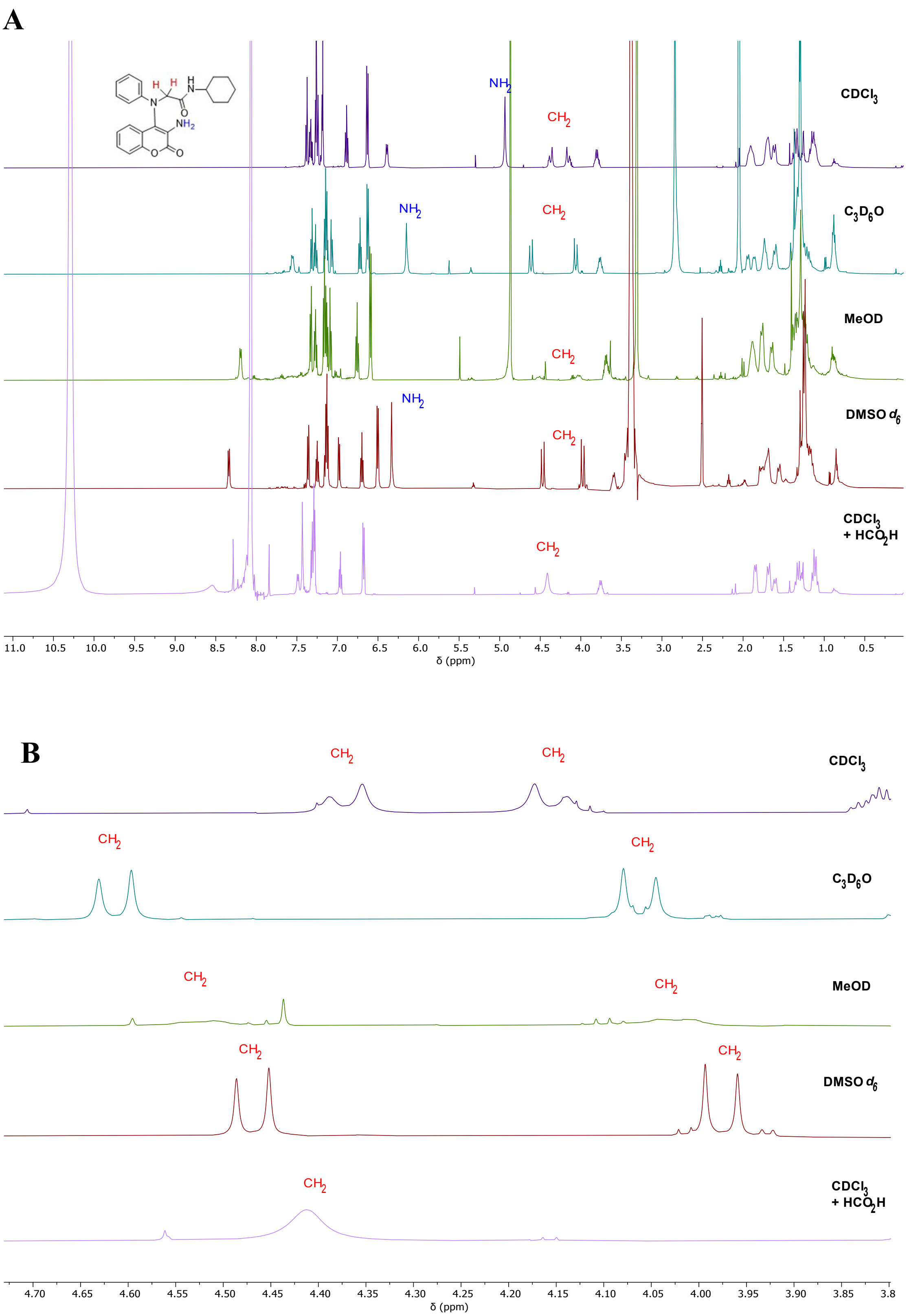

Figure S4. **A**. Stacked ^{1}HNMR spectra of compound **10** in different solvents. **B**. Expansion of the area showing the signals corresponding to the diastereotopic protons.

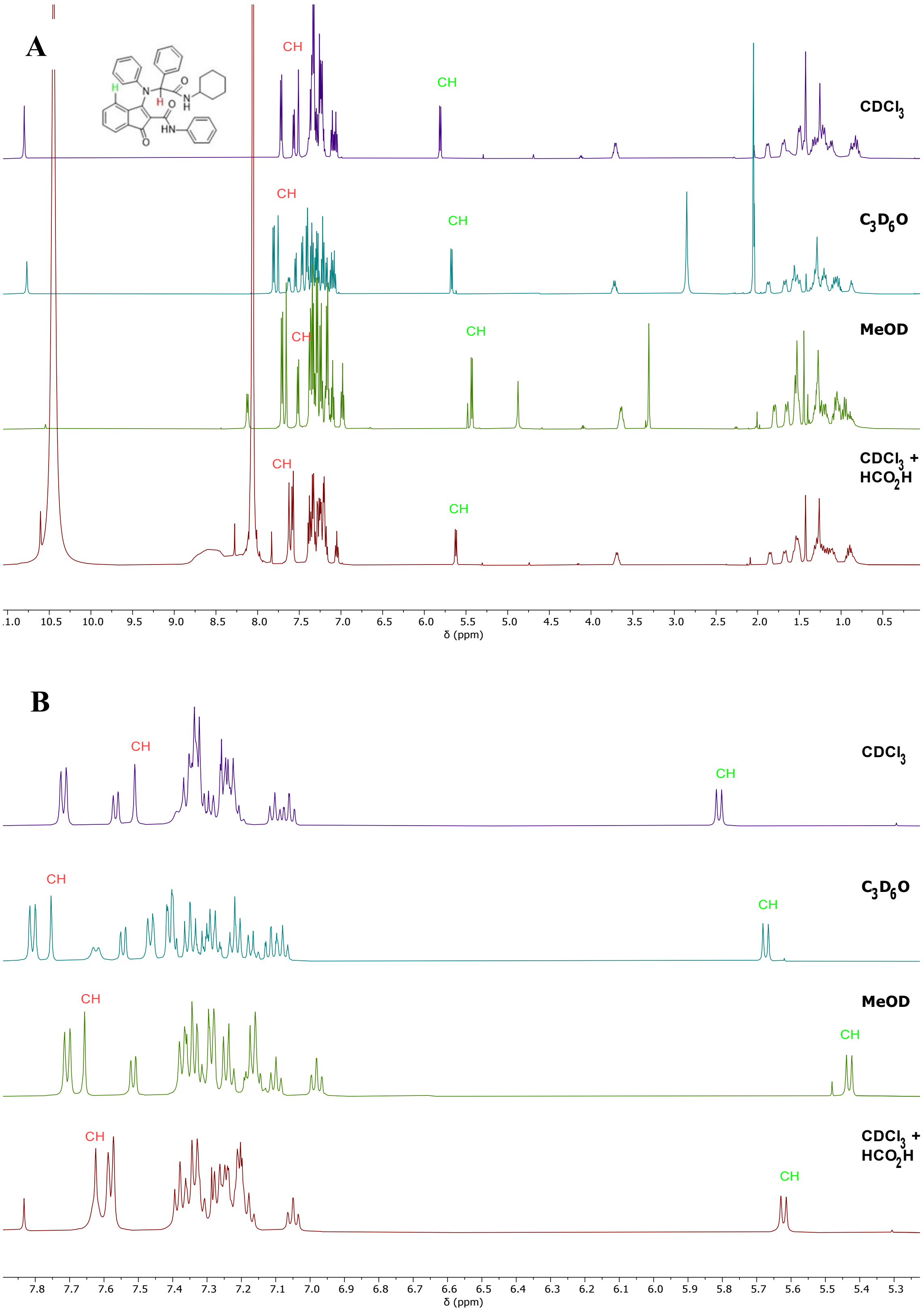

Figure S5. **A**. Stacked ^{1}HNMR spectra of compound **8** in different solvents. **B**. Expansion of the area showing the signals corresponding to the proton on the stereogenic carbon.

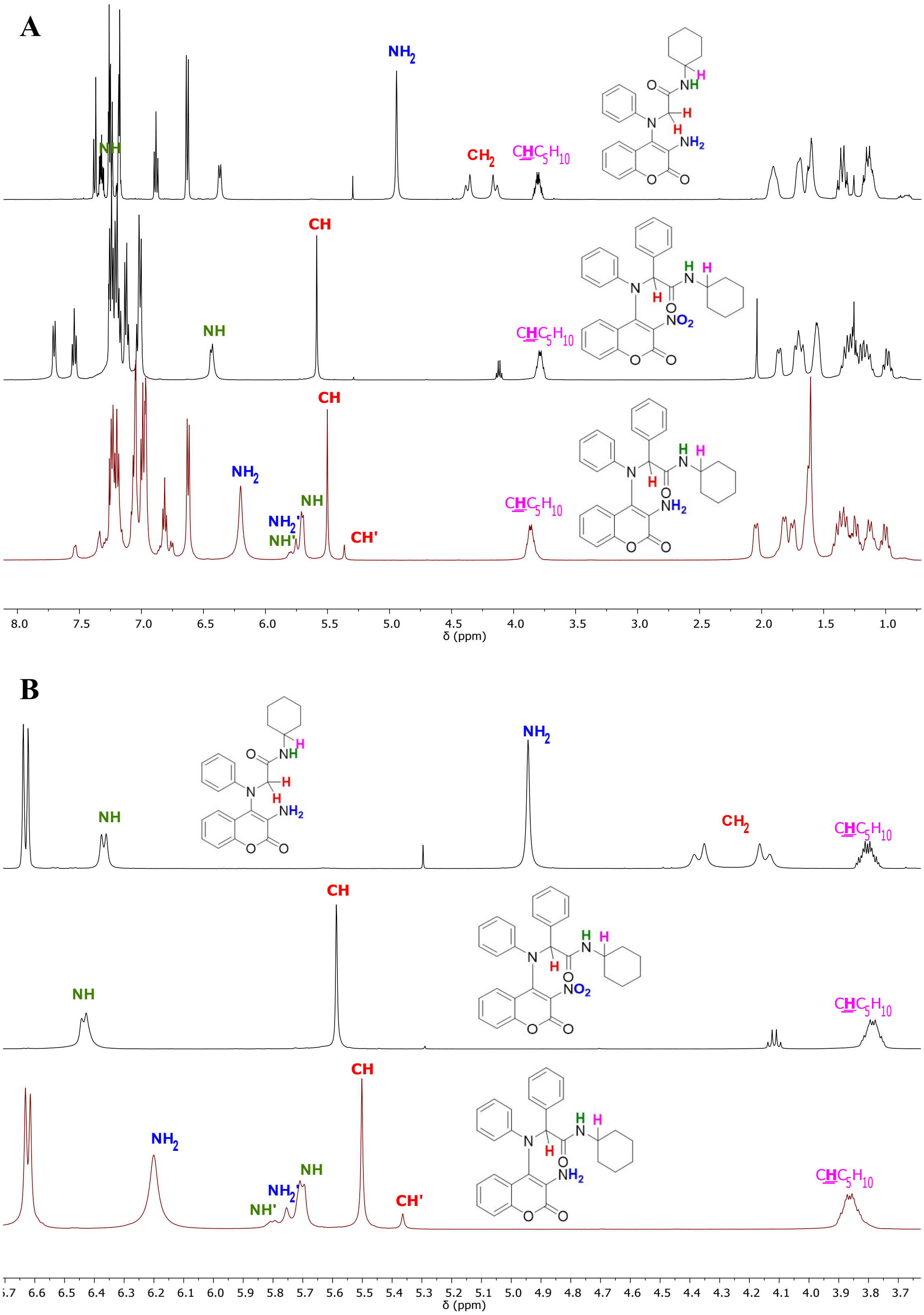

Figure S6. **A**. ^{1}HNMR spectra of compounds **7**, **9** and **10**. **B**. Expansion of the area showing the signals corresponding to the protons on the carbon α to the amide group.

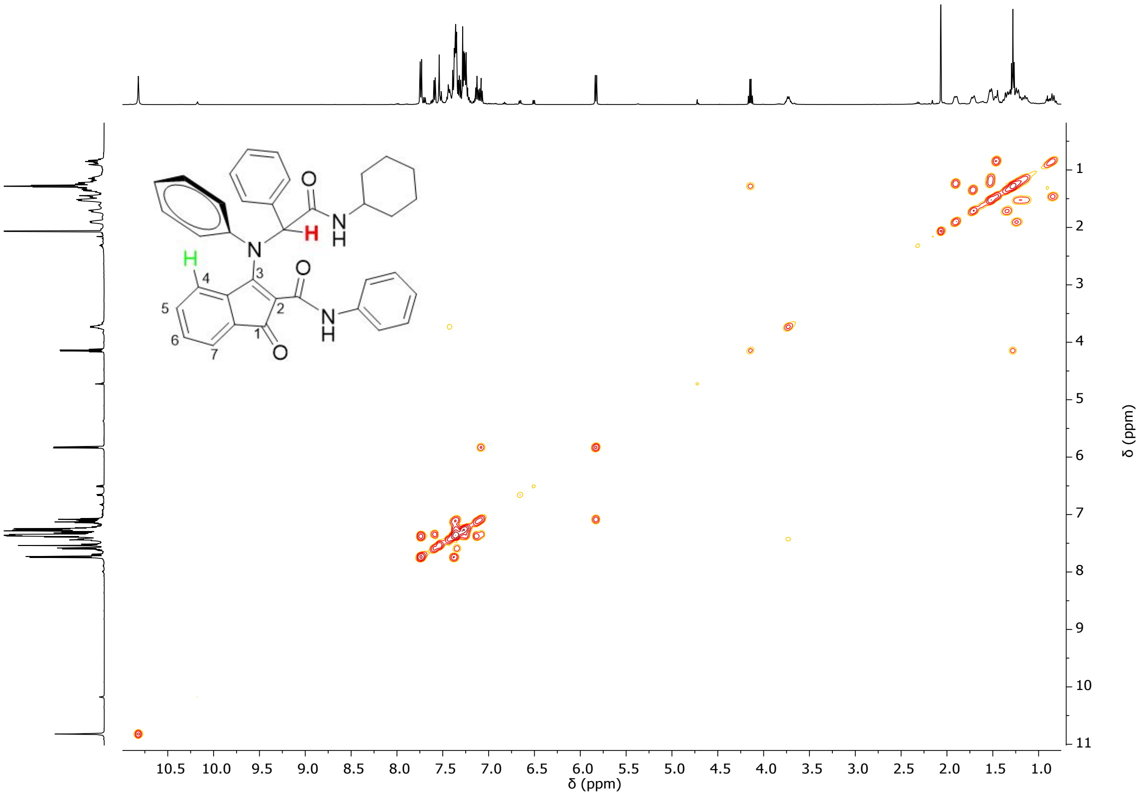

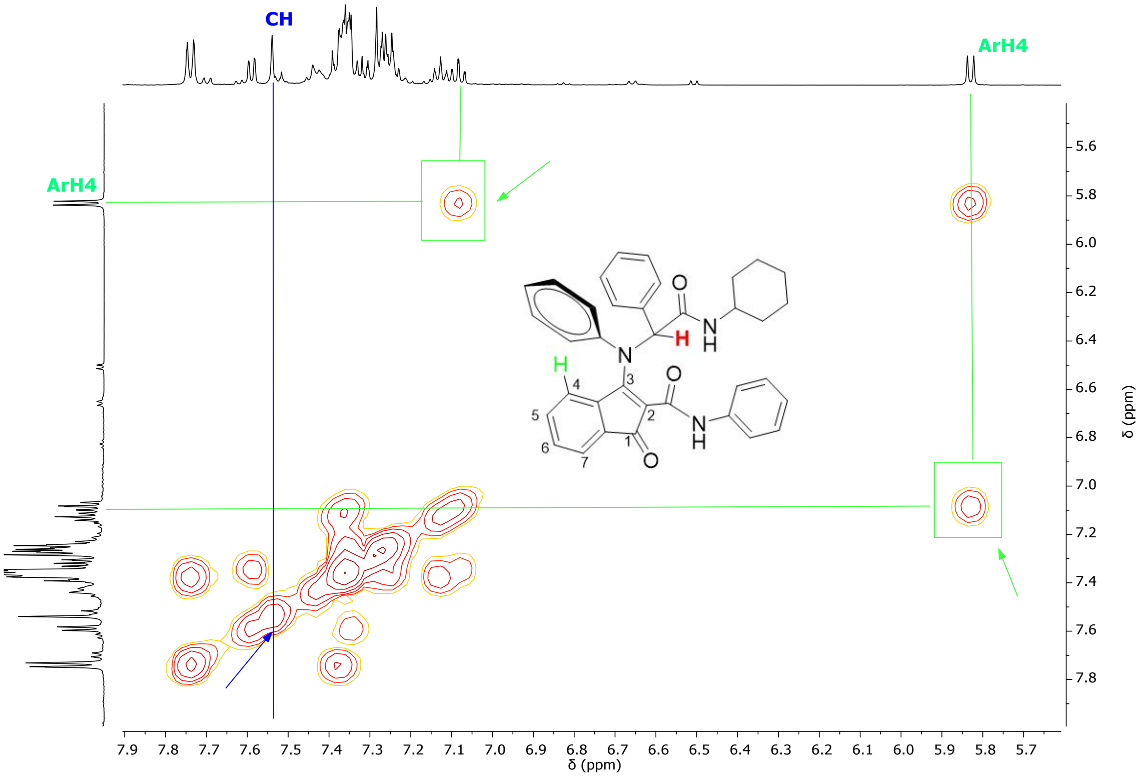

Figure S7. ^{1}H-^{1}H-COSY spectrum of 3-((2-(cyclohexylamino)-2-oxo-1-phenylethyl)(phenyl)amino)-1-oxo-*N*-phenyl-1*H*-indene-2-carboxamide (**8**) and expansion.

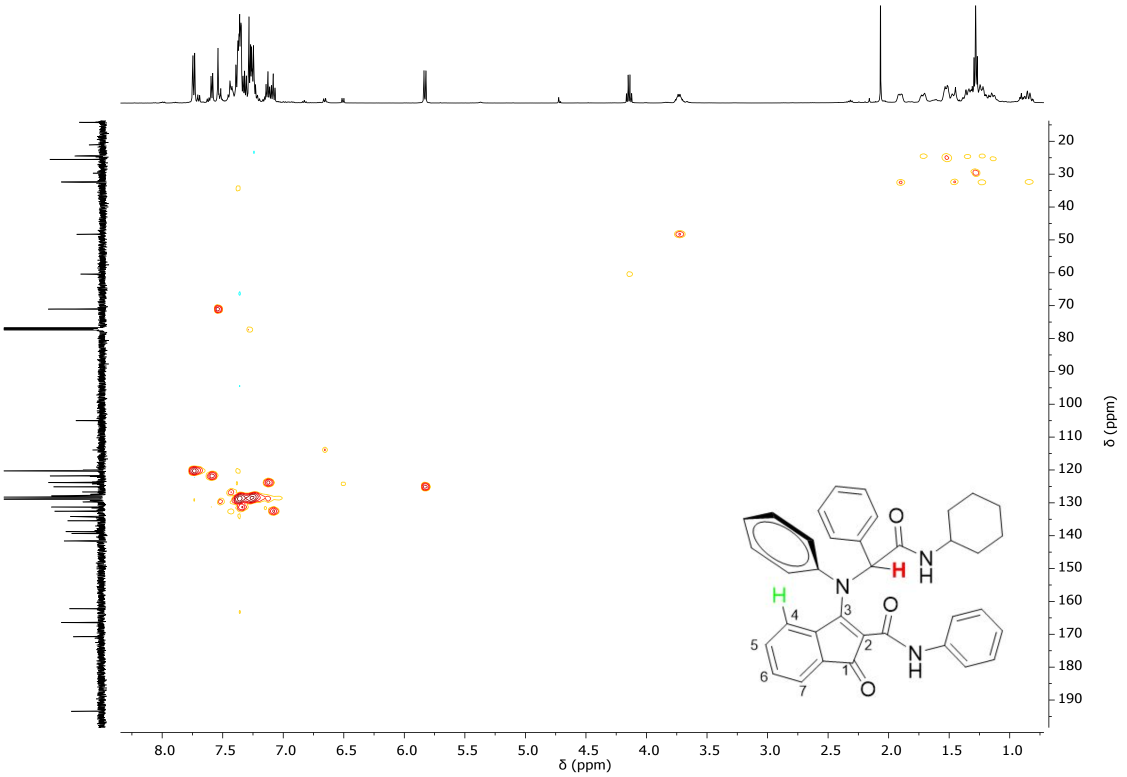

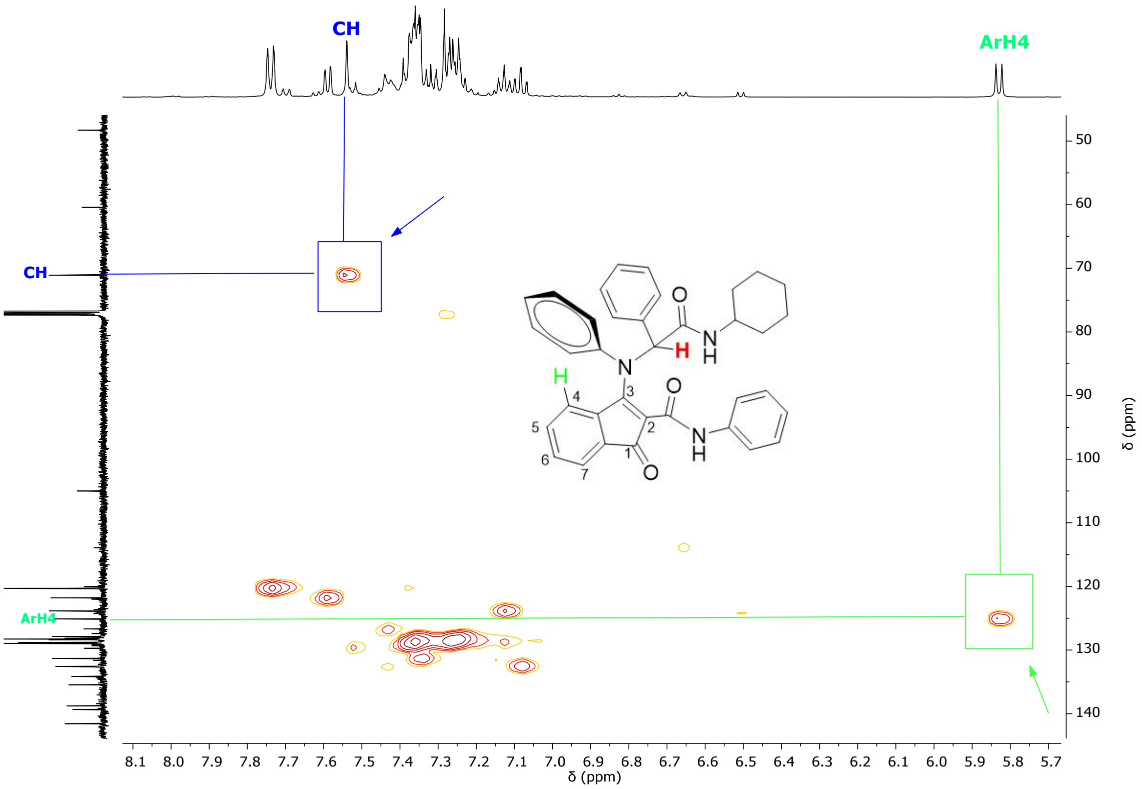

Figure S8. ^{13}C-^{1}H-HSQC spectrum of 3-((2-(cyclohexylamino)-2-oxo-1-phenylethyl)(phenyl)amino)-1-oxo-*N*-phenyl-1*H*-indene-2-carboxamide (**8**) and expansion.

Calculation of the experimental rotational energy barriers.

Rotational barrier for adduct 7

Rotational energy barriers, ΔG_{major} and ΔG_{minor}, were obtained by applying Erying's equations as modified by Shanan-Atidi and Bar-Eli for unequal populations of rotamers. [4-5]

$$\Delta G_{major} = 4{,}57 * Tc * (10{,}62 + \log\left(\frac{X}{2\pi(1-\Delta P)}\right) + \log\left(\frac{Tc}{\Delta\nu}\right)) \qquad \text{(a)}$$

$$\Delta G_{minor} = 4{,}57 * Tc * (10{,}62 + \log\left(\frac{X}{2\pi\,(1+\Delta P)}\right) + \log(\frac{Tc}{\Delta\nu})) \qquad \text{(b)}$$

Tc is the coalescence temperature, determined by TV-NMR spectroscopy. Figure S2 shows that the signals belonging to the two rotamers coalesce at 110.15 ºC (373.15 K) in the solvent 1,1,2,2-tetrachloroethane-*d2*. **Δν** is the frequency difference between the signals of stereogenic proton (C-H) from both rotamers at room temperature, that was determined to be 63.5 Hz. ***ΔP*** is the difference between the populations of the conformers and is defined as follows:

$$\Delta P = P_{major} - P_{minor} \qquad \text{(c)}$$

P_{major} and P_{minor} are obtained from the ^{1}HNMR spectrum in 1,1,2,2- tetrachloroethane-*d2* at room temperature, by integrating the signal of the stereogenic C-H corresponding to each rotamer. P_{major} was 0.85 and P_{minor} was 0.15, resulting in a *ΔP* of 0.7. Finally, the last parameter required in equations (a) and (b) is $\boldsymbol{X}$, which is defined as $X = 2\tau\pi\Delta\nu$, derived from the mean lifetime, τ, and the chemical shift difference Δν between conformers. Moreover, X can also be calculated from *ΔP*, applying equation (d):

$$\Delta P = \left(\frac{X^2-2}{3}\right)^{\frac{3}{2}} * \frac{1}{X} \qquad \text{(d)}$$

Given *ΔP* = 0.7, X was calculated to be 2.53. With all parameters determined, the rotational energy barrier for the interconversion of rotamers was determined using equations (a) and (b):

$$\Delta G_{major} = 18.33\ kcal/mol$$
$$\Delta G_{minor} = 17.04\ kcal/mol$$

Rotational barrier for adduct 10

Since compound (**10**) exists as two enantiomeric conformers, the experimental rotational barrier between them was estimated using the Eyring's equation assuming equal populations[5-6]:

$$\Delta G = 4{,}57 * Tc * (10{,}62 + \log\left(\frac{Tc}{kc}\right)) \qquad \text{(e)}$$

Tc is the coalescence temperature (343,15 K), which is derived from the TV NMR experiment, and **kc** is the rate constant at **Tc**, that is experimentally determined by equation (f)

$$kc = \frac{\pi \Delta v}{\sqrt{2}} \qquad \text{(f)}$$

Where **Δv** represents the frequency difference between the signals of the diastereotopic protons, which was found to be 150 Hz, yielding a *kc* value of 333.22 s^{-1}.

Applying equations (e) and (f) the experimental rotational barrier for compound **10** was found to be $\Delta G = 16{,}29\ kcal/mol$.

Computational methods.

All calculations were performed with GAUSSIAN16.[7] Geometries were optimised in the gas-phase using the B3LYP/6-31G(d) level of theory.[8-11] Frequency calculations (at 298.15 K) at the same level of theory were used to confirm the nature of all stationary points as minima and provided values to compute free energies. A systematic conformational search was performed for **6**, **7**, **8** and **10** at the same level, testing various attempts to find the lowest energy conformers for each structure. NMR single point calculations (GIAO)[12-16] were performed on these geometries at the B3LYP+G(d,p) level of theory with chloroform as solvent employing the Solvent Model Density (SMD) within the Self-Consistent Reaction Field method SCRF.[17]

^{1}HNMR computed chemical shift.

For each structure, **6** and **8**, all conformers within a 10 kcal/mol energy window were subjected to a Boltzmann-weighted averaging strategy based on their computed relative free energies at 298.15 K. The relative populations were computed as:

$$\frac{P_i}{P_j} = e^{\frac{-(E_i - E_j)}{RT}} \qquad \text{(e)}$$

Where:

P_i = Population of conformer i relative to lowest energy conformer j.
E_i, E_j = computed free energies in J/mol,
R = molar gas constant (8.314510 J/(mol K))
T = 298.15 K

The relative populations were then converted to weighting factors, that is, percentages. The computed chemical shifts of each nucleus were then multiplied by these percentages to produce a population-weighted average. The conformational search, within a 10 kcal/mol window, yielded 54 conformers for compound **6** and 125 for compound **8**, respectively. These conformers were weighted by their energies and used to compute the NMR shifts of all protons, as detailed below.

Computed chemical shifts are typically scaled empirically to eliminate systematic errors originating from various sources. These scaling factors are usually determined by comparing computed NMR data with known experimental chemical shifts from large molecular databases. The factors, which include the slope and intercept of a best-fit line, are specific to each computational level of theory used. For calculations of ^{1}HNMR chemical shifts in structures **6** and **8** we have employed the database generated by Tantillo and coworkers (http://cheshirenmr.info).[18]

The selected method for obtaining quality chemical shift predictions at reasonable cost was the GIAO method in SCRF/SMD chloroform at the B3LYP/6-31+G(d,p) level of theory for NMR calculations, with an initial optimization at B3LYP/6-31G(d) level.

The specific scaling factors for the method employed in this work were slope (-1.0591) and intercept (31.643), which were applied on the computed NMR isotropic shielding constants by means of the following equation:

$$\delta = \frac{b - \sigma}{-m} \quad \text{(f)}$$

Where:

δ = computed chemical shift relative to TMS in ppm.
σ = computed isotropic shielding constant.
m = slope (-1.0591).
b = intercept (31.643).

Non-covalent Interactions (NCI) calculations.

The Non-Covalent Interaction (NCI) index[19-20] is a visualisation tool based on electron density (ρ) and its derivatives. It allows for the identification of non-covalent interactions using the reduced density gradient. These interactions can be either favourable or unfavourable. The NCI index was calculated at the B3LYP/6-31G(d) level for the minimum energy conformers of the four species (**6**, **7**, **8**, and **10**). The resulting electronic density map facilitates the visualization of various interactions between the functional groups within the molecules, with attractive interactions displayed in blue and repulsive interactions in red.

6A

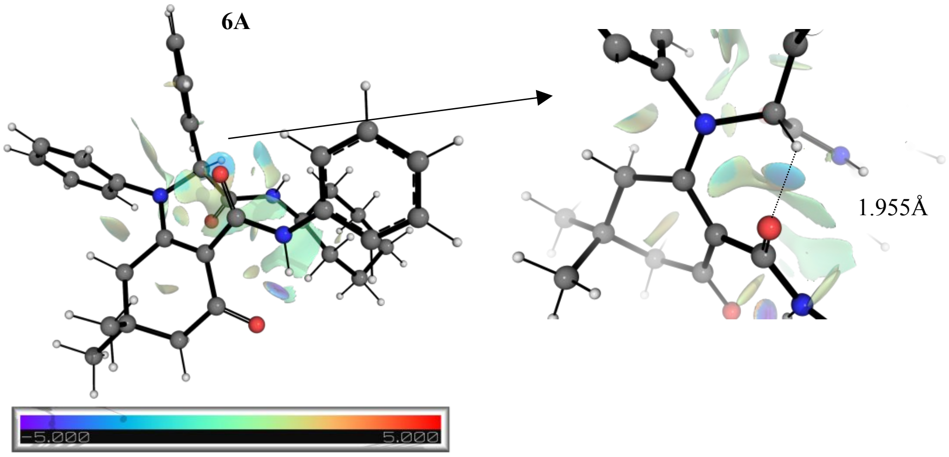

6B

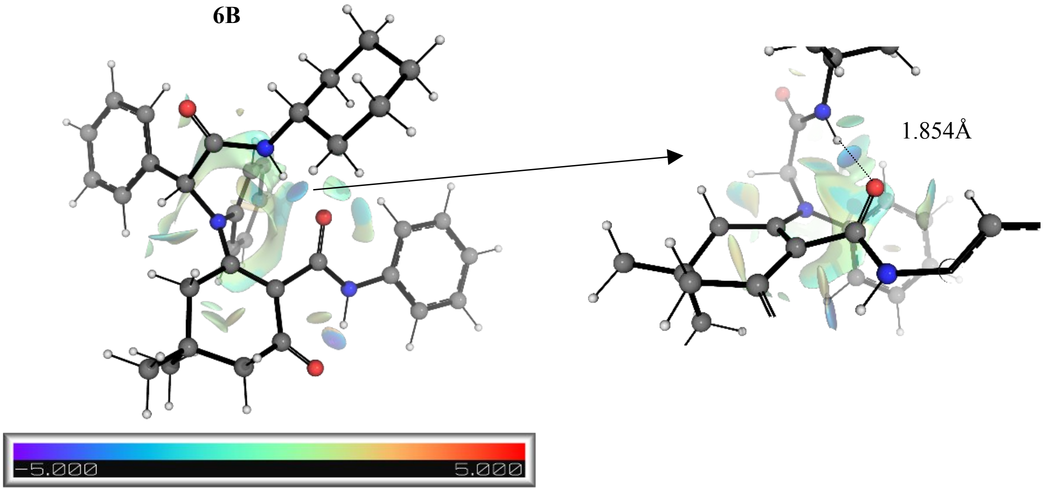

7

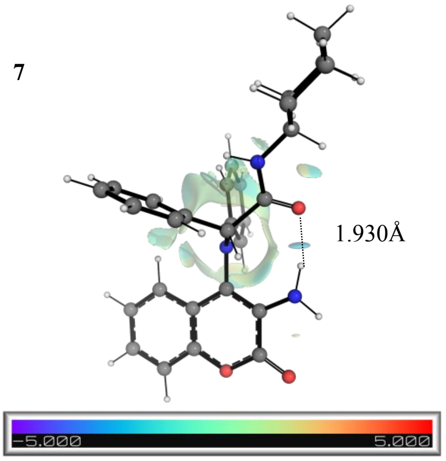

10

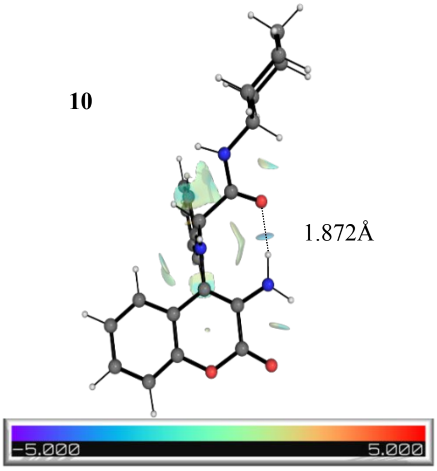

8

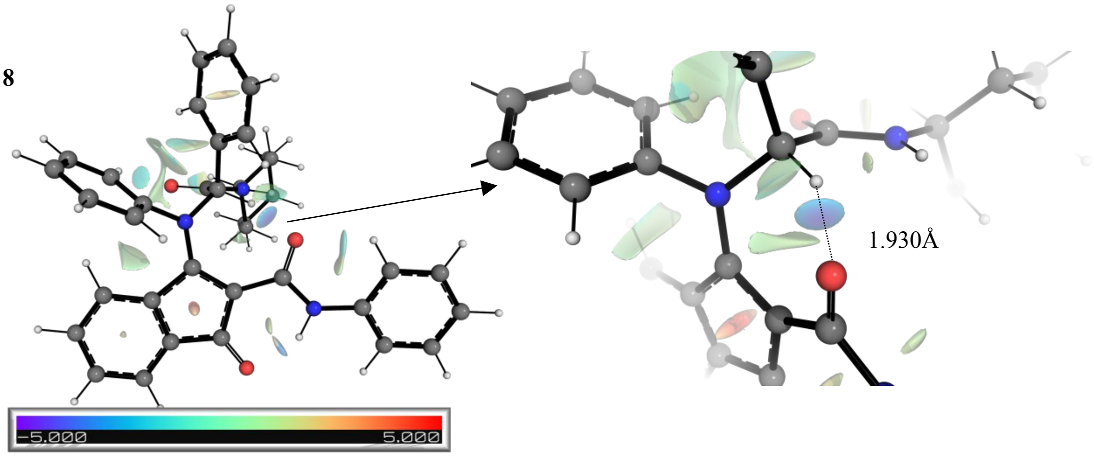

Figure S9. NCI plots for the compound **6** (the two most stable conformers **6A** and **6B**), and most stable conformers of compounds **7**, **10** and **8** with scale colours from attractive (blue) to repulsive (red) interactions.

Minima and maxima geometries for compounds 7 and 10

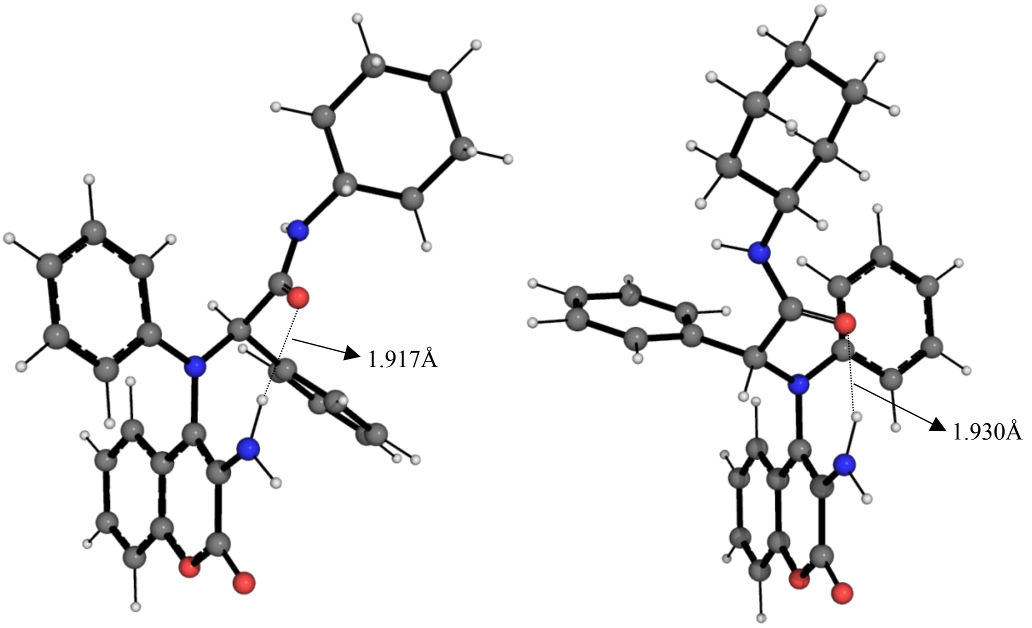

Figure S10. Structures of the two most stable rotamers of compound **7**.

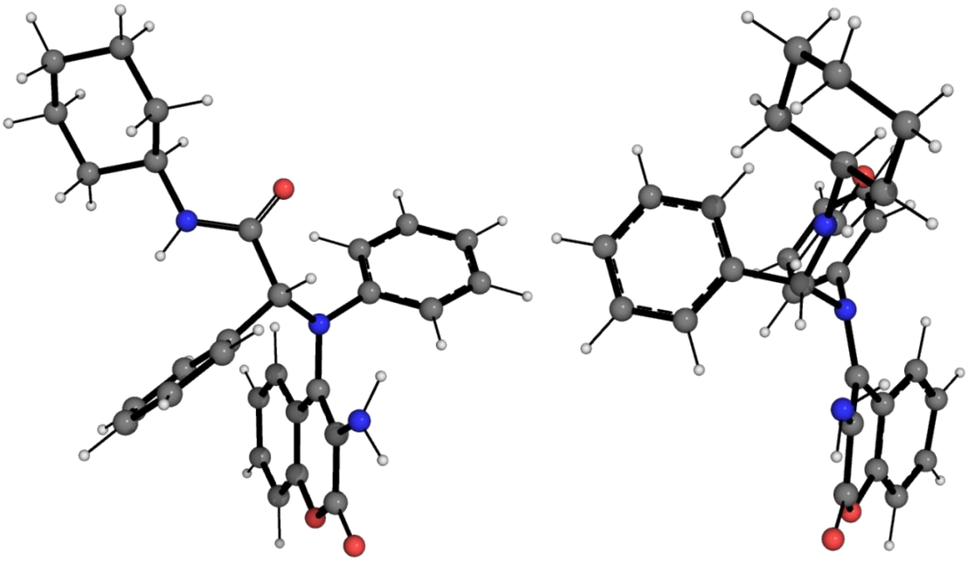

Figure S11. Structures of the geometry of the two maxima found for **7**.

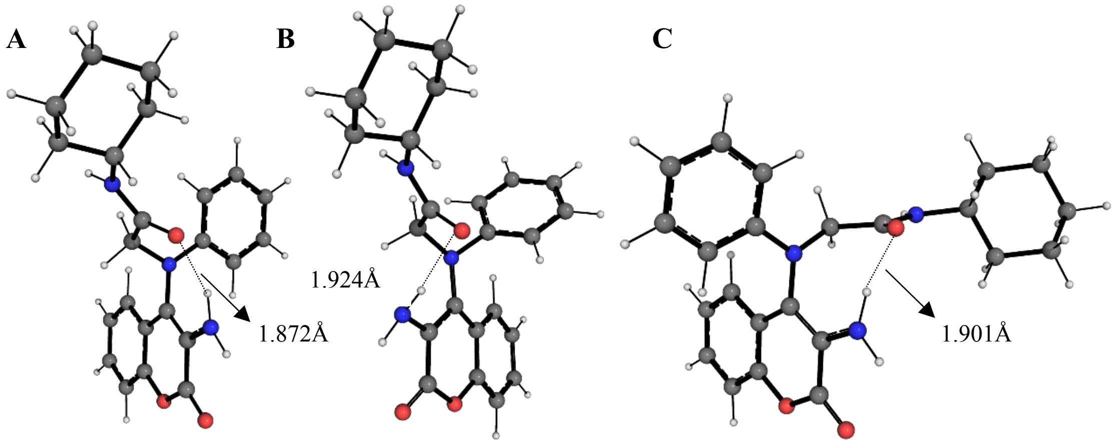

Figure S12. Geometries of the two minima (**A** and **C**) and maximum (**B**) found for **10**.

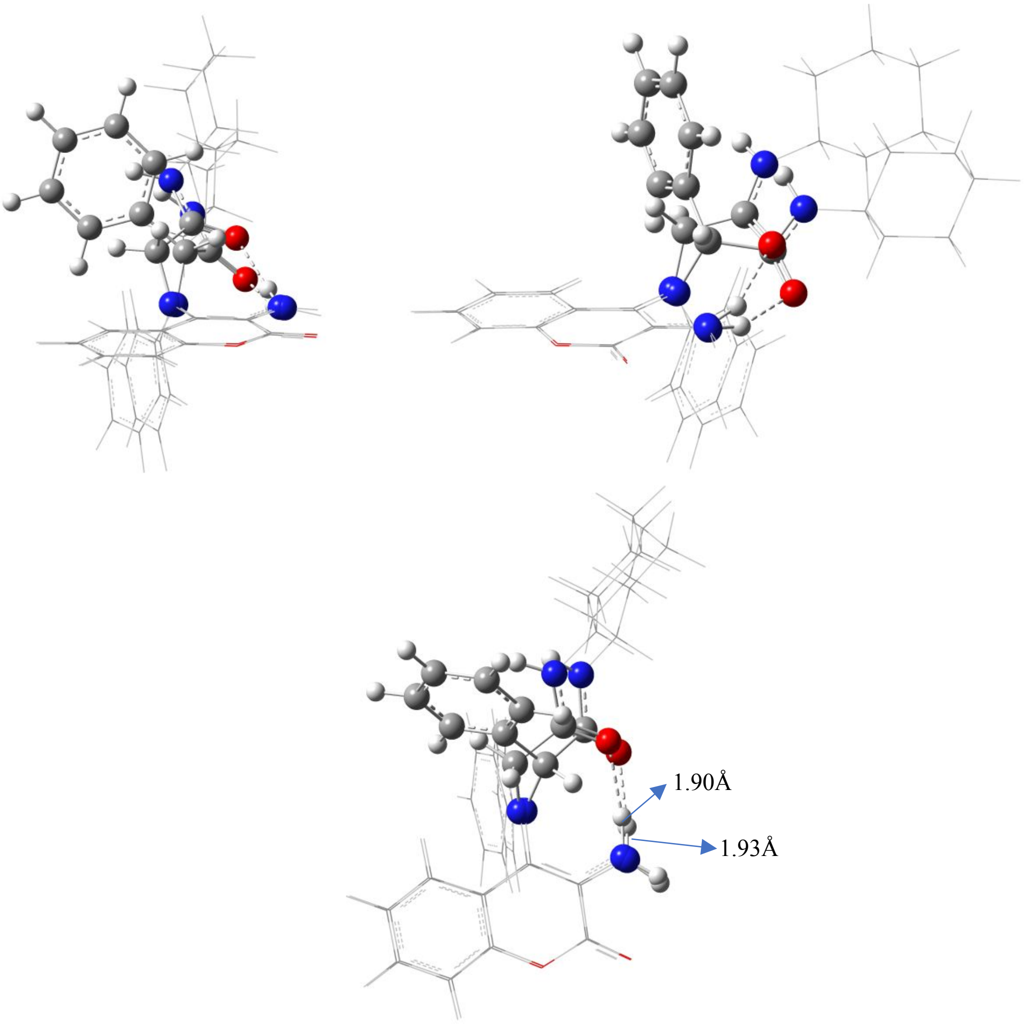

Figure S13. Overlapped optimised geometries of compounds **7** and **10**, visualised from different perspectives.

Matrices XYZ for computationally derived structures of compounds 6-10.

The xyz matrices for the computationally most stable structures of compounds **6**, **7**, **8** and **10**, alongside the matrices for the maxima of **7** and **10**.

XYZ matrix for computationally most stable conformers.zip

References

1. Bornadiego, A.; Neo, A. G.; Marcos, C. F., Synthesis of chromeno[3,4-b]piperazines by an enol-Ugi/reduction/cyclization sequence. *Molecules* **2021,** *26* (5), 1287-1287. doi: http://dx.doi.org/10.3390/molecules26051287

2. Neo, A. G.; Castellano, M. T. G.; Ramiro, J. L.; Marcos, C. F., Amide-stabilized enols in the enol-Ugi reaction: A five-component synthesis of triamides. *Chem Proc* **2022,** *8*, 102. doi: http://dx.doi.org/10.3390/ecsoc-25-11665

3. Ramiro, J. L.; Neo, A. G.; Marcos, C. F., Amide-derived enols in enol–Ugi reactions: Expanding horizons for peptidomimetic scaffold synthesis. *Organic & Biomolecular Chemistry* **2024**. doi: http://dx.doi.org/10.1039/d4ob01216j

4. Shanan-Atidi, H.; Bar-Eli, K. H., A convenient method for obtaining free energies of activation by the coalescence temperature of an unequal doublet. *The Journal of Physical Chemistry* **1970,** *74* (4), 961-963. doi: http://dx.doi.org/10.1021/j100699a054

5. Frank, J. H.; Powder-George, Y. L.; Ramsewak, R. S.; Reynolds, W. F., Variable-temperature 1H-NMR studies on two C-glycosylflavones. *Molecules* **2012,** *17* (7), 7914-7926. doi: http://dx.doi.org/10.3390/molecules17077914

6. Friebolin, H., *Basic one- and two-dimensional NMR spectroscopy*. 5th completely rev. and enlarged ed.; WILEY-VCH: Weinheim, 2011.

7. Frisch, M. J.; Trucks, G. W.; Schlegel, H. B.; Scuseria, G. E.; Robb, M. A.; Cheeseman, J. R.; Scalmani, G.; Barone, V.; Petersson, G. A.; Nakatsuji, H.; Li, X.; Caricato, M.; Marenich, A. V.; Bloino, J.; Janesko, B. G.; Gomperts, R.; Mennucci, B.; Hratchian, H. P.; Ortiz, J. V.; Izmaylov, A. F.; Sonnenberg, J. L.; Williams; Ding, F.; Lipparini, F.; Egidi, F.; Goings, J.; Peng, B.; Petrone, A.; Henderson, T.; Ranasinghe, D.; Zakrzewski, V. G.; Gao, J.; Rega, N.; Zheng, G.; Liang, W.; Hada, M.; Ehara, M.; Toyota, K.; Fukuda, R.; Hasegawa, J.; Ishida, M.; Nakajima, T.; Honda, Y.; Kitao, O.; Nakai, H.; Vreven, T.; Throssell, K.; Montgomery Jr., J. A.; Peralta, J. E.; Ogliaro, F.; Bearpark, M. J.; Heyd, J. J.; Brothers, E. N.; Kudin, K. N.; Staroverov, V. N.; Keith, T. A.; Kobayashi, R.; Normand, J.; Raghavachari, K.; Rendell, A. P.; Burant, J. C.; Iyengar, S. S.; Tomasi, J.; Cossi, M.; Millam, J. M.; Klene, M.; Adamo, C.; Cammi, R.; Ochterski, J. W.; Martin, R. L.; Morokuma, K.; Farkas, O.; Foresman, J. B.; Fox, D. J. *Gaussian 16 rev. C.01*, Wallingford, CT, 2016.

8. Becke, A. D., Density-functional thermochemistry. III. The role of exact exchange. *The Journal of Chemical Physics* **1993,** *98* (7), 5648-5652. doi: http://dx.doi.org/10.1063/1.464913

9. Parker, W. L.; Rathnum, M. L.; Johnson, J. H.; Wells, J. S.; Principe, P. A.; Sykes, R. B., Aerocyanidin, a new antibiotic produced by chromobacterium violaceum. *J. Antibiot.* **1988,** *41*, 454-460.

10. Vosko, S. H.; Wilk, L.; Nusair, M., Accurate spin-dependent electron liquid correlation energies for local spin density calculations: A critical analysis. *Can. J. Phys.* **1980,** *58* (8), 1200-1211. doi: http://dx.doi.org/10.1139/p80-159

11. Stephens, P. J.; Devlin, F. J.; Chabalowski, C. F.; Frisch, M. J., Ab initio calculation of vibrational absorption and circular dichroism spectra using density functional force fields. *The Journal of Physical Chemistry* **2002,** *98* (45), 11623-11627. doi: http://dx.doi.org/10.1021/j100096a001

12. London, F., Théorie quantique des courants interatomiques dans les combinaisons aromatiques. *Journal de Physique et le Radium* **1937,** *8* (10), 397-409. doi: http://dx.doi.org/10.1051/jphysrad:01937008010039700

13. McWeeny, R., Perturbation theory for the fock-dirac density matrix. *Physical Review* **1962,** *126* (3), 1028-1034. doi: http://dx.doi.org/10.1103/PhysRev.126.1028

14. Ditchfield, R., Self-consistent perturbation theory of diamagnetism. *Mol. Phys.* **1974,** *27* (4), 789-807. doi: http://dx.doi.org/10.1080/00268977400100711

15. Wolinski, K.; Hinton, J. F.; Pulay, P., Efficient implementation of the gauge-independent atomic orbital method for NMR chemical shift calculations. *J. Am. Chem. Soc.* **2002,** *112* (23), 8251-8260. doi: http://dx.doi.org/10.1021/ja00179a005

16. Cheeseman, J. R.; Trucks, G. W.; Keith, T. A.; Frisch, M. J., A comparison of models for calculating nuclear magnetic resonance shielding tensors. *The Journal of Chemical Physics* **1996,** *104* (14), 5497-5509. doi: http://dx.doi.org/10.1063/1.471789

17. Marenich, A. V.; Cramer, C. J.; Truhlar, D. G., Universal solvation model based on solute electron density and on a continuum model of the solvent defined by the bulk dielectric constant and atomic surface tensions. *J Phys Chem B* **2009,** *113* (18), 6378-96. doi: http://dx.doi.org/10.1021/jp810292n

18. Lodewyk, M. W.; Siebert, M. R.; Tantillo, D. J., Computational prediction of 1H and 13c chemical shifts: A useful tool for natural product, mechanistic, and synthetic organic chemistry. *Chem Rev* **2012,** *112* (3), 1839-62. doi: http://dx.doi.org/10.1021/cr200106v

19. Contreras-García, J.; Johnson, E. R.; Keinan, S.; Chaudret, R.; Piquemal, J. P.; Beratan, D. N.; Yang, W., Nciplot: A program for plotting noncovalent interaction regions. *Journal of Chemical Theory and Computation* **2011,** *7*, 625−632. doi: http://dx.doi.org/10.1021/ct100641a

20. Johnson, E. R. K., S.;Mori-Sańchez,P.;Contreras-García,J.; Cohen, A. J.; Yang, W., Revealing noncovalent interactions. . *J. Am. Chem. Soc.* **2010,** *132*, 6498−6506. doi: http://dx.doi.org/10.1021/ja100936w
